# Differential transcript expression profiles of susceptible and resistant pigeonpea cultivars at an early time point during *Fusarium udum* infection

**DOI:** 10.3389/fgene.2022.1009127

**Published:** 2022-10-05

**Authors:** Sanatan Ghosh, Arnab Purohit, Anjan Hazra, Aloleca Mukherjee, Anirban Bhar, Sumanti Gupta, Rituparna Kundu Chaudhuri, Dipankar Chakraborti

**Affiliations:** ^1^ Department of Genetics, University of Calcutta, Kolkata, West Bengal, India; ^2^ EVA.4 Unit, Faculty of Forestry and Wood Sciences, Czech University of Life Sciences Prague, Prague, Czechia; ^3^ Post Graduate Department of Botany, Ramakrishna Mission Vivekananda Centenary College (Autonomous), Kolkata, West Bengal, India; ^4^ Department of Botany, Rabindra Mahavidyalaya, Hooghly, West Bengal, India; ^5^ Department of Botany, Barasat Govt. College, Kolkata, West Bengal, India

**Keywords:** comparative transcriptomics, *Fusarium* wilt, next generation sequencing, fungal invasion, gene ontology

## Introduction

Pigeonpea [*Cajanus* cajan (L.) Millspaugh] is ranked seventh among the legume crops, in terms of production, and is grown in arid and semiarid tropical regions of Asia, Africa, the Caribbean region, Latin America, and Australia. It is rich in vegetable protein (20%–22%), and its global productivity is nearly 5.012 million tonnes (FAO 2020). Pigeonpea is mostly grown as a field crop and as a backyard crop in more than 80 countries all over the world (Sameer Kumar et al., 2017). It is cultivated on 5.62 million hectares of land across the world, and India contributes 64% (2.85 million tons) of global production (Saxena et al., 2017). Pigeonpea is the second-most significant crop legume in India, mostly consumed as “dal.” Seeds are an important source of protein for humans, whereas stems and leaves are used as fuel and animal feed. Vascular wilt caused by *Fusarium udum* (Butler) is the most damaging disease in pigeonpea and results in an annual loss of approximately 470,000 tons of grain in India (Saxena et al., 2017). *F. udum* is a soilborne, mitosporic, and necrotrophic fungus without known sexual stages in its lifecycle (Agrios 2008). *F. udum* produces three types of asexual spores, namely, thick-walled chlamydospores, 2–6 celled macroconidia, and 1–2 celled microconidia. The most frequently and abundantly produced spores are microconidia which are also found inside the infected host’s vascular system. Macroconidia are primarily found on the surface of infected and dead host plants in sporodochia-like groupings. The old mycelium of the pathogen produces chlamydospores which can survive in the soil for a very long period (Purohit et al., 2017).

In pigeonpea, *F. udum*-mediated vascular wilt occurs at the early or late flowering, podding, or even seedling stages (Choudhury 2010). Xylem vessel clogging is an important phenomenon that leads to wilt in infected pigeonpea. Infected xylem vessels of roots and stems become clogged with spores and mycelia of the pathogen, as well as polysaccharides produced by it. Additionally, xylem parenchyma cells of the infected root are induced by the pathogen to divide excessively. This situation combined with weaker and thinner xylem vessel walls causes a reduced diameter or complete collapse (Agrios 2008). *F. udum* also secretes toxins that are eventually carried to the leaves through xylem vessels, reducing chlorophyll synthesis and disrupting leaf cell membrane permeability and thereby leading to transpirational water loss. At the later stage of infection, the host plant shows wilting, yellowish leaf color, interveinal necrosis, and eventually death. Based on the growth stage of the infected plant and the severity of the wilt, yield loss can be up to 100% (Reddy et al., 1990).

Management of *F. udum* wilt was achieved through chemical treatment of seeds, crop rotation, and the development of biocontrol agents. Although wilt-resistant cultivars have been established through breeding programs dealing with pigeonpea, their usage was restricted due to the presence of variability among the pathogens and the existence of location-specific pathogenic races. As a result, the breakdown of resistance was evident in resistant cultivars (Dhar et al., 2012). Under this situation, resistance (R) gene pyramiding could be a promising strategy to develop resistant genotypes. However, all the R genes were not mapped, and the lack of information leads to very complex time-consuming marker-assisted breeding programs. Previous research works were also focused on the characterization of *F. udum* isolates from India, taken from various geographic regions, using molecular variability, cultural characteristics, and pathogenesis in pigeonpea. Thirteen *F. udum* isolates from India were identified through cultural and morphological methods and molecular fingerprinting techniques (Dhar et al., 2012; Purohit et al., 2017). Four variants and seven phylogenetic groups were established from the RAPD and AFLP data, respectively. The pathogenesis of nine infective isolates was studied, and the timing of the invasion of the pathogen, clogging of vascular bundles, drooping of leaves, and complete wilting were demonstrated in pigeonpea by the present group. Invasion of all nine isolates of *F. udum* in healthy pigeonpea root was identified at 24–36 hours post-inoculation (HPI), determined through anatomical, morphological, and biochemical studies of the infected root (Purohit et al., 2017). At 36 HPI (i.e., invasion stage), cDNA-AFLP-mediated comparative transcriptomics study on *F. udum* inoculated root of wilt-susceptible (ICP 2376) and wilt-resistant pigeonpea cultivars (ICP 8863) along with their mock inoculated controls were performed by Purohit et al. (2021). Among all differentially expressed transcript-derived fragments (TDFs), many were identified to be involved in disease resistance or tolerance mechanisms. The identified important defense responsive pathways were pathogen-triggered immunity, effector-triggered immunity, reactive oxygen species-mediated signaling, salicylic acid- and jasmonic acid-mediated defense responses, cell wall remodeling, vascular development and pattering, and abscisic acid-mediated responses. These pathways were found to be activated during pathogen attacks and played crucial roles in defense responses (Purohit et al., 2021).

Next-generation sequencing (NGS)-mediated transcriptome profiling is a convenient method to recognize the mechanism of host defense during *Fusarium* wilt. It is very useful to identify differentially regulated genes and novel signaling pathways associated with wilt-resistance mechanisms. RNA-seq-based NGS studies have been conducted during *Fusarium* wilt in the model system, *Arabidopsis thaliana*, and various legumes, such as common bean (*Phaseolus vulgaris*), pea, chickpea, soybean, and pigeonpea. Soren et al. (2021) reported RNA-seq data through comparative analysis in Bahar (susceptible) and KPL-44 (resistant) pigeonpea cultivars at late infection stages such as 72 HPI and 96 HPI in response to *F. udum* attack. They considered the untreated susceptible and resistant plants at 0 h as controls for both infection time points.

On the basis of our previous report, it was found that at the early stage of *F. udum* infection, maximum disease-responsive pathways were altered in pigeonpea (Purohit et al., 2021). Accordingly, the present study was designed to perform NGS-based comparative transcriptomic profiling of *F. udum*-induced transcripts at the early (36 HPI) infection stage in pigeonpea through an RNA-seq technique to accomplish a thorough understanding of the differentially expressed genes. This will help to obtain comprehensive knowledge of the tolerant or resistant mechanisms, underlying metabolic pathways influenced by *F. udum*, and putative regulatory genes which are involved in complex spatiotemporal regulation for wilt resistance.

## Materials and methods

### Cultivars of pigeonpea

Seeds of wilt-susceptible ICP 2376 and wilt-resistant ICP 8863 cultivars were collected from International Crops Research Institute for the Semi-Arid Tropics (ICRISAT), Hyderabad, Telangana, India. After surface sterilization of seeds, susceptible and resistant seedlings were grown in soilrite at 22–25°C, 35%–40% humidity, and a 16-h photoperiod (Purohit et al., 2021).

### *F. udum* isolate

*F. udum* isolate MTCC 2204 was collected from the Microbial Type Culture Collection and Gene Bank of Institute of Microbial Technology, Chandigarh, India. A single colony of MTCC 2204 was inoculated in potato dextrose broth (PDB) and potato dextrose agar (PDA) media and permitted to incubate at 25°C. After 8–10 days of growth, sporulation was checked.

### Inoculation of resistant and susceptible cultivars with the MTCC 2204 pathogenic isolate

Seeds of the ICP 2376 and ICP 8863 cultivars were surface sterilized using 0.05% mercuric chloride (HgCl_2_) and germinated in a sterile cotton bed. After germination, seedlings were carefully established in soilrite-filled pots. All the plants were maintained at 35%–40% humidity, and 22–25°C with a 16-h photoperiod in a plant growth chamber. After 14–15 days, inoculation of seedlings was performed with the *F. udum* MTCC 2204 isolate as described previously (Purohit et al., 2017).

M1 isolate was cultured in PDB, and conidia were harvested from two-week-old suspension culture. The conidial concentration was adjusted to 1 × 10^6^ ml^−1^ in PDB. Two hundred grams of sand:chickpea meal (9:1) was mixed thoroughly with 50 ml of the MTCC 2204 spore suspension. This mixture was incubated in dark for 14 days at 25°C. Then, this sand:chickpea meal infested with *F. udum* was mixed thoroughly with a sand:soilrite (1:1) mix. The mix was filled in pots, and both susceptible and resistant seedlings were transplanted. A sand:soilrite (1:1) mix without fungal inoculum was used for transplanting control plants from both cultivars. Fifteen plants, each of ICP 2376 and ICP 8863, were inoculated with MTCC 2204 isolate and another 15 plants, each of ICP 2376 and ICP 8863, were used as negative controls. All of the seedlings were maintained under the previously mentioned growth conditions. Each experiment was repeated three times.

### RNA isolation and double-stranded cDNA preparation

Roots of noninoculated susceptible (NIS), noninoculated resistant (NIR), MTCC 2204 inoculated susceptible (IS), and inoculated resistant (IR) plants were obtained at 36 hours post-inoculation (HPI). The roots of three plants (biological replications) of each noninoculated control and pathogen-inoculated treatment were pooled together for RNA isolation. Root tissues (500 μg) were washed thoroughly using distilled water and frozen immediately in liquid nitrogen. Frozen root tissues were crushed into a fine powder using mortar–pestle. Total RNA isolation was performed using TRIzol reagent (Ambion, Thermo Fisher Scientific, Massachusetts, United States) as mentioned in the protocol provided by the manufacturer. RNA isolated from three experimental replications was pooled for each control and treatment. Qualitative and quantitative assessments of the isolated and pooled total RNA were performed by measuring the absorbance using a nanodrop spectrophotometer followed by agarose gel electrophoresis. From the total RNA, mRNA purification was done using a Qiagen Oligotex mRNA Minikit (Qiagen, Hilden, Germany). Subsequently, a SMARTer PCR-cDNA synthesis kit (Clontech Laboratories, Inc., Dalian, China) was used to prepare double-stranded cDNA, from each of the purified poly-A mRNA samples (500 ng) following the instructions provided by the manufacturer.

### Next-generation sequencing

The Illumina NextSeq500 platform was used for transcriptome sequencing. Double-stranded cDNA libraries were incorporated into an Illumina chamber for the generation of clusters. After obtaining the Qubit concentration for cDNA libraries, 2 × 150 bp paired-end sequencing was performed through the sequencing by synthesis method. Paired-end sequencing allowed the fragmented templates to be sequenced in both forward and reverse directions which were used for transcriptome sequencing. After sequencing, FASTQ files were generated for each sample.

### Sequence quality analysis

Next-generation sequencing analysis was performed on cDNA samples collected at 36 hours post-inoculation with MTCC 2204. The quality control for raw paired-end sequence reads was examined using the FastQC version 0.11.3 (http://www.bioinformatics.babraham.ac.uk/projects/fastqc/) program (Supplementary Material S1). The pair-end reads with <30 PHRED quality score were shortlisted, and >30 paired-end reads along with all the unpaired reads were eliminated. Following this step, processing of raw Illumina reads was performed using Trimmomatic software (version 0.32)-based analysis (Bolger et al., 2014) for removal of universal adapters and trimming of low-quality bases incorporated inside and also at the 3′ end region of the sequences.

### De novo transcriptome assembly

De novo assembly of the Illumina processed datasets was performed using Trinityrnaseq_r20140717 software, and further filtration was performed to develop the Cluster of Genes (COGs) assembly. Trinityrnaseq_r20140717 (Grabherr et al., 2011) was used for de novo assembly after correction of all possible errors of the processed sequenced reads. Scaffolding of assembled contigs was carried out using the SSPACE software program (https://github.com/nsoranzo/sspace_basic) (Boetzer et al., 2011). Gap closer was performed to remove the polyN inserted during scaffolding using the GapCloser software (https://github.com/BGI-Qingdao/TGS-GapCloser) (Xu et al., 2019).

### Transcript annotation

Candidate coding regions with the generated transcript sequences were identified using the TRANSDECODER tool, and processed transcripts were annotated using NCBI BLAST N 2.303 with the DNA and protein sequences of *C. cajan*, *Glycine max*, *Phaseolus vulgaris*, *Vigna radiata*, *Lotus Japonicas*, *Medicago truncatula*, and *Arabidopsis thaliana*. The fungal sequence contaminations present in the transcriptome data were removed. Then, the purified sequences were used as the reference to map the reads of all four samples to generate the gene matrix with the normalized fragments per kilobase of transcript per million (FPKM) values. Based on the matrix and the provided conditions, the sequences were further filtered and grouped into the corresponding categories. Gene Ontological classification (GO) was performed with the aid of the Blast2GO software program (Gotz et al., 2008), and pathway annotation of all the transcripts was done using KEGG pathway analysis software.

### Identification of differentially expressed genes

The DESeq package (version 1.8.1) (http://www-huber.embl.de/users/anders/DESeq/) was used to perform differential gene expression analysis and a total number of up-, down- and neutrally regulated transcripts were identified between noninoculated vs. inoculated resistant cultivar (NIR vs IR) and noninoculated vs. inoculated susceptible cultivar (NIS vs IS). By comparing the base mean expression values of the inoculated samples with the matching control samples, fold change was obtained. The number of common and uniquely occurred transcripts across all conditions in both the cultivar was represented using Venny 2.1.0 software (https://bioinfogp.cnb.csic.es/tools/venny/index.html). Fold-change distributions of the assembled transcripts were represented using volcano plots in R (version 4.2.1) along with the corresponding log-transformed *p*-values. Statistically significant outcomes from all the differentially expressed transcripts were filtered using a 0.05 *p*-value limit.

## Results and discussion

### NGS raw read data

The total numbers of raw reads were 28.39 million (m), 28.31, 35.74, and 26.25 m for noninoculated resistant (NIR), noninoculated susceptible (NIS), inoculated resistant (IR), and inoculated susceptible (IS), respectively. Following Trimmomatic filtering, 27.2, 26.95, 4.35, and 25.36 m clean reads were obtained from NIR, NIS, IR, and IS, respectively. These datasets were used for further downstream analysis. All the RNA-seq raw reads have been deposited in the NCBI Sequence-Read Archive (SRA) database under the bio project PRJNA782089. Table 1 shows the transcriptome data summary with corresponding NCBI-SRA accession numbers (Supplementary Materials S1, S2).

**TABLE 1:** Statistical summary of the transcriptome data output with their NCBI-SRA accession numbers.

Sample identity	Genotype	Sample type	Read type	Total read count	PHRED quality score	GC content (%)	Reads mapped in pairs	Accession
NIR	ICP 8863	noninoculated resistant	150 × 2 Paired end	28,395,501	phred33	44	28,060,243 (98.82%)	SRR16990781
IR	ICP 8863	Inoculated resistant	150 × 2 Paired end	35,740,467	phred33	49	35,303,808 (98.78%)	SRR16990783
NIS	ICP 2376	noninoculated susceptible	150 × 2 Paired end	28,311,348	phred33	47	27,895,856 (98.53%)	SRR16990780
IS	ICP2376	Inoculated susceptible	150 × 2 Paired end	26,252,636	phred33	50	26,004,461 (99.05%)	SRR16990782

NIR, noninoculated resistant; IR, inoculated resistant; NIS, noninoculated susceptible; IS, inoculated susceptible; NCBI, national center for biotechnology information.

### De novo transcriptome assembly of processed data

De novo transcriptome assembly of processed reads into transcripts generated 322,688 in total number with a maximum transcript length of 24,582 base pairs (bp) and a minimum length of 300 bp. The total transcript length was found to be 295,830,572 bp with 0% non-ATGC characteristics. After filtration, COG statistics showed 222,874 transcripts generated after clustering with 57 very large (Transcripts >10 Kb), 53,932 large (Transcripts >1 Kb), 115,970 medium (Transcripts≥500), and 222,874 small (Transcripts≥300) transcripts. The total identified transcript length after filtration was 190,178,549 bp without any non-ATGC characteristics in the matrix (Table 2).

**TABLE 2:** Analysis of de novo assembly transcriptome data.

Transcript statistics	Transcriptome de novo assembly statistics	COG’s statistics	Transcripts >500bp statistics
Sample name	Pooled samples	Pooled samples	Pooled samples
Tool used	Trinity 25	Trinity 25	Trinity 25
Hash length	25	25	25
Transcripts generated	3,22,688	2,22,874	1,15,970
Maximum transcript length	24,582	24,582	24,582
Minimum transcript length	300	300	300
Average transcript length	916.8 ± 875.9	853.3 ± 837.1	1,291.5 ± 971.3
Median transcript length	656	406	1,659.5
Total transcript length	29,58,30,572	19,01,78,549	14,97,79,299
Total number of non-ATGC characteristics	0	0	0
Transcripts ≥300 b	322,688	222,874	115,970
Transcripts ≥500 b	182,013	115,970	115,970
Transcripts >1 Kb	90,111	53,932	53,932
Transcripts >10 Kb	85	57	57

### Transcript annotation and Gene Ontological analysis

**FIGURE 1:**
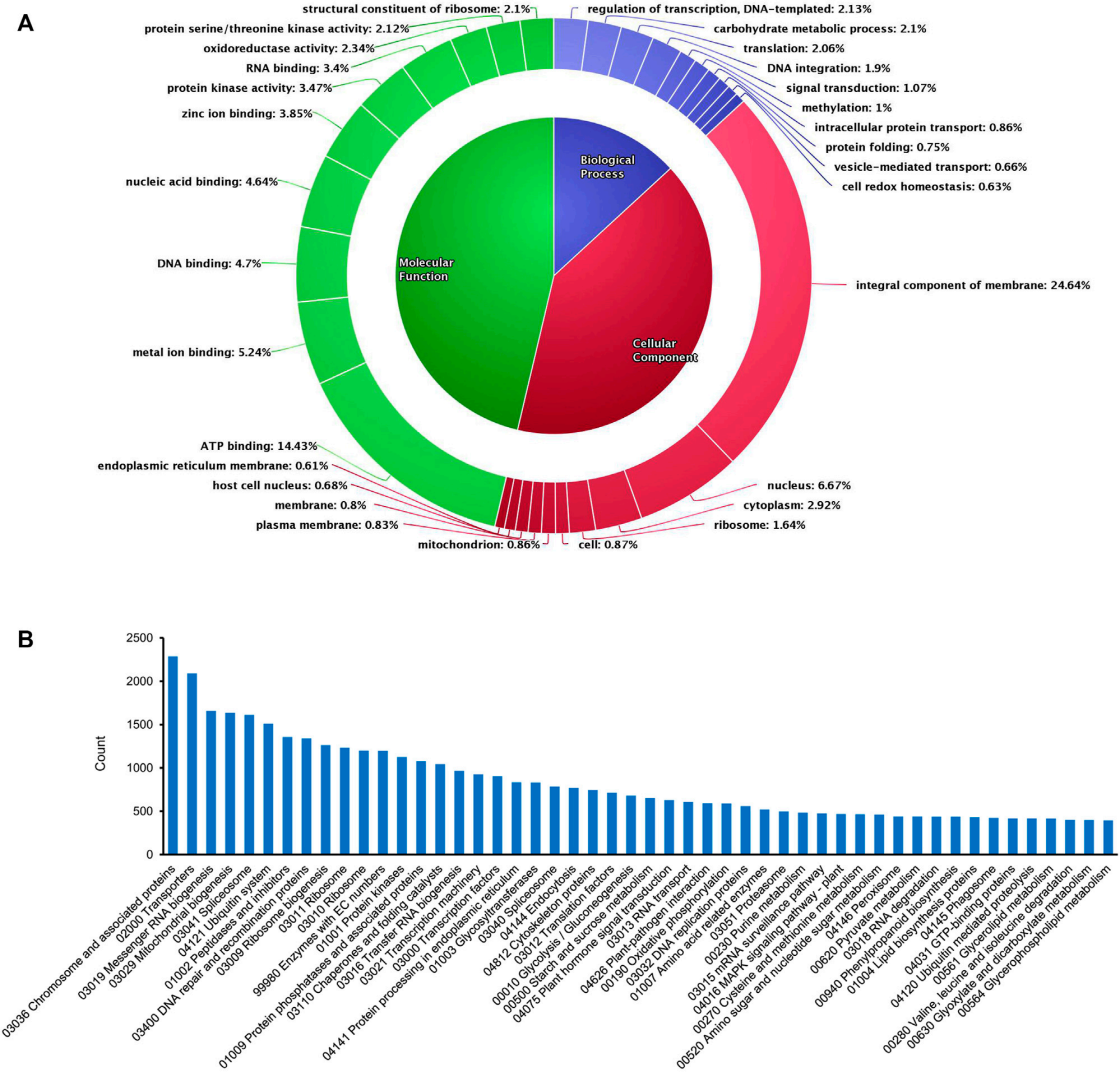
Overview of GO and KEGG enrichment analysis of the de novo assembled transcripts in the present study. **(A)** Gene Ontology chart showing the distribution of various transcripts. **(B)** Top 50 pathways observed in KEGG pathway analysis.

De novo assembly enabled us to acquire maximum transcripts from pigeonpea as annotations were done using the database of the *C. cajan* genome. However, in the case of transcripts with no possible match against *C. cajan*, *Glycine max* genome was considered, as it was the closest relative to pigeonpea, followed by *Phaseolus vulgaris*, *Vigna radiata*, *Lotus Japonicas*, *Medicago truncatula*, and *Arabidopsis thaliana*. After the annotation program, out of 98,466 total transcripts, 70,981 transcripts were annotated and the remaining 27,485 were unannotated. Clustering of total annotated transcripts was performed using various database searches (NCBI, UNIPROT, and pFAM) based on their gene ontology, which revealed varying distributions of transcripts across biological processes (13.6%), molecular functions (46.29%), and cellular components (40.52%) (Figure 1A). Among the biological processes, transcripts involved in the regulation of transcription (2.13%), carbohydrate metabolic processes (2.1%), translation (2.06%), DNA integration (1.9%), signal transduction (1.07%), methylation (1%), intracellular protein transport (0.86%), protein folding (0.75%), vesicle-mediated transport (0.66%), and cell redox homeostasis (0.63%) were observed. In the case of cellular components, transcripts of integral components of membrane and signaling (24.64%) were mostly observed. In addition, transcripts with cellular locations in the nucleus (6.67%), cytoplasm (2.92%), ribosome (1.64%), mitochondrion (0.86%), and plasma membrane (0.83%) were expressed. Transcripts associated with molecular functions were found in nearly half of the total number. This group consisted of transcripts of ATP binding (14.43%), metal ion binding (5.24%), DNA binding (4.7%), nucleic acid binding (4.64%), zinc ion binding (3.85%), protein kinase related (3.47%), RNA binding (3.4%), oxidoreductase (2.34%), serine/threonine kinase (2.12%), and structural constituent of ribosome (2.1%). KEGG pathway analysis of the transcriptome data showed that 64,768 annotated transcripts were involved in 204 different pathways under six major groups: brite hierarchies, cellular processes, environmental information processing, genetic information processing, metabolism, and organismal systems. Figure 1B shows the top 50 pathways observed in the KEGG enrichment analysis.

### Analysis of differentially expressed transcripts

**FIGURE 2:**
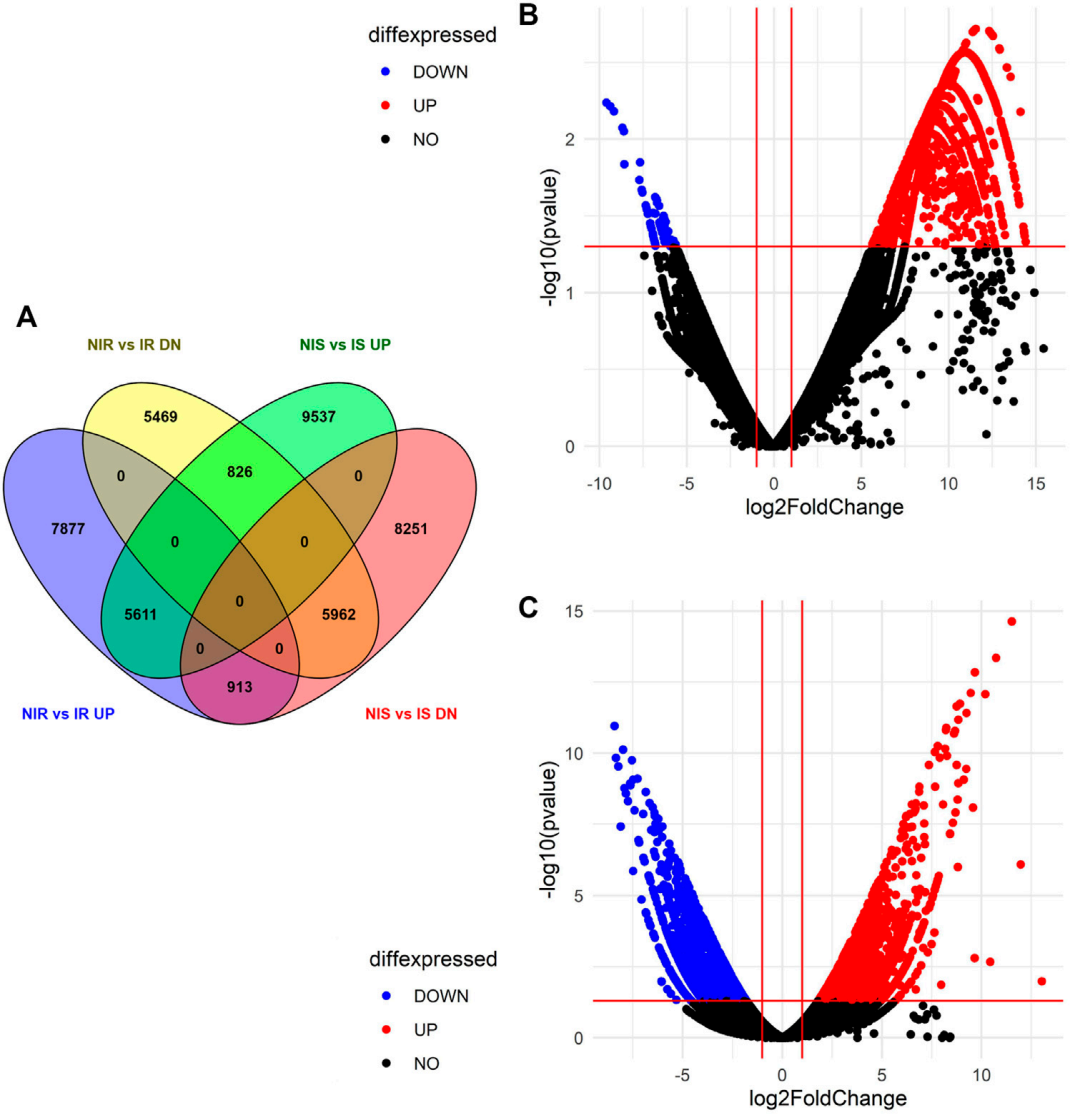
Differential expressed genes identified in both the susceptible and resistant cultivars. **(A)** Venny 2.1.0 software analysis of differential expression of genes in NIR vs IR and NIS vs IS. **(B)** Volcano plot representing DEG data in NIS vs IS. **(C)** Volcano plot representing DEG data in NIR vs IR.

Differential expression of gene study between noninoculated vs. inoculated resistant cultivar (NIR vs IR) revealed that a total of 75,799 transcripts were expressed in both samples, out of which 14,401 upregulated, 12,257 downregulated, and 49,141 neutrally regulated transcripts were present. A total of 5,153 and 6,538 transcripts were found only in NIR and IR, respectively. Similarly, the differential expression of genes between noninoculated vs. inoculated susceptible cultivar (NIS vs IS) depicted a total of 67,634 expressed transcripts with 15,974 up-, 15,126 down-, and 36534 neutrally regulated transcripts. A total of 10,022 and 19,979 transcripts are only expressed in NIS and IS, respectively. Venny's analysis of differentially regulated genes showed that 5,611 (12.6%) upregulated and 5,962 (13.4%) downregulated transcripts were common in both NIR vs IR and NIS vs IS. A total of 7,877 (17.7%) and 5,469 (12.3%) transcripts were exclusively up- and downregulated in NIR vs IR, respectively. Similarly, a total of 9,537 (21.5%) and 8,251 (18.6%) transcripts were exclusively up- and downregulated in NIS vs IS, respectively. Interestingly, 826 (1.9%) transcripts were found to be upregulated in NIS vs IS but downregulated in NIR vs IR, and 913 (2.1%) transcripts were found to be upregulated in NIR vs IR but downregulated in NIS vs IS (Figure 2A). After visualizing the *p* (0.05) values and the distribution of fold change of the DEGs in the NIS vs IS and NIR vs IR groups, they were presented by volcano plot (Figures 2B,C).

## Conclusion

From the gene ontological as well as the differential gene expression data of both control and inoculated wilt-susceptible and -resistant transcriptomes, stress- and defense-related genes such as peroxidase73, RPM1, RIN4, RPP8, RPS2, MLO-like protein, wound-induced protein WIN, WRKY70, WRKY33, PR1, TMV-resistant protein, WAT1, MYB46, CESA, and many more such genes can be considered proposed leads for future molecular-based research of pigeonpea. These identified important putative genes along with their associated pathways, activated during the attack of *F. udum* can be investigated through time kinetics studies to understand signaling molecules for host–pathogen interactions and varied metabolic pathways that have crucial roles in inherent resistance mechanisms. Further research can be extended to validate the expression of these genes, as well as the identified disease susceptibility or resistance pathways through functional genomics approaches. This will provide improved knowledge of the resistance mechanisms in pigeonpea during *F. udum* wilt which can be utilized for genomics-assisted breeding programs, genome editing, and biotechnological improvement.

## Data Availability

The datasets presented in this study can be found in online repositories. The names of the repository/repositories and accession number(s) can be found in the NCBI Sequence-Read Archive (SRA) database under the bio-project PRJNA782089.
