# Supplementary material for: Differential transcript expression profiles of susceptible and resistant pigeonpea cultivars at an early time point during Fusarium udum infection
Source: Front Genet. 2022 Oct 5;13:1009127. doi: 10.3389/fgene.2022.1009127 (PMC9581238; doi:10.3389/fgene.2022.1009127)
Supplement: Supplementary file 1 [file Table1.DOCX]

**S2.** **FastQC report of generated RNA-seq data after trimmomatic filtering (A-H).**


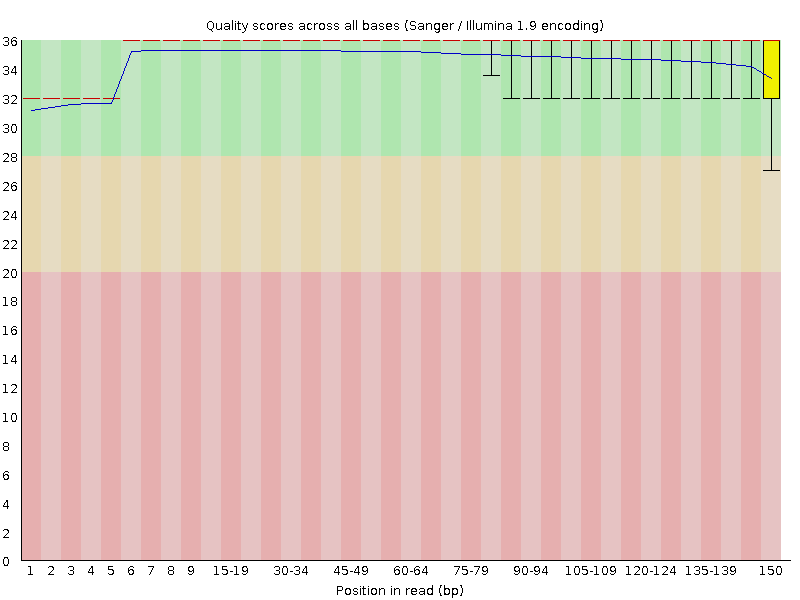

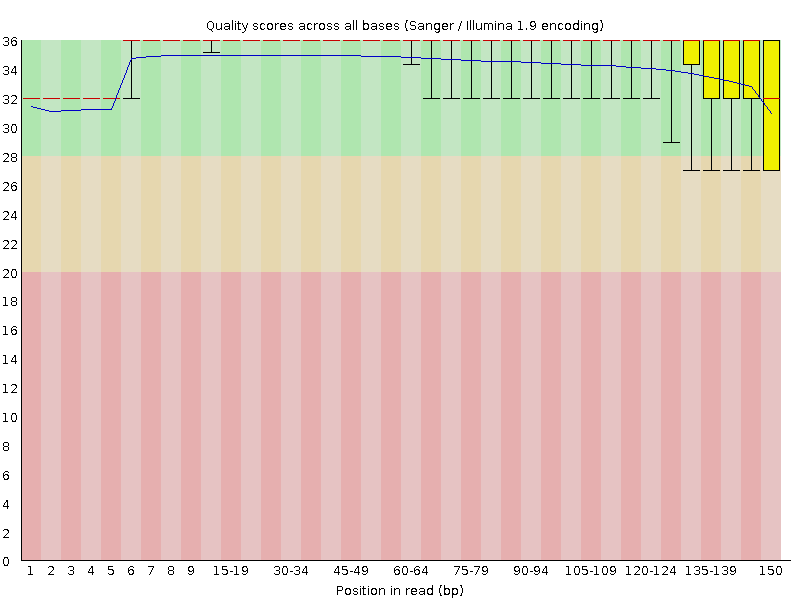


B. FastQC IR Reverse

1. FastQC IR Forward Forward


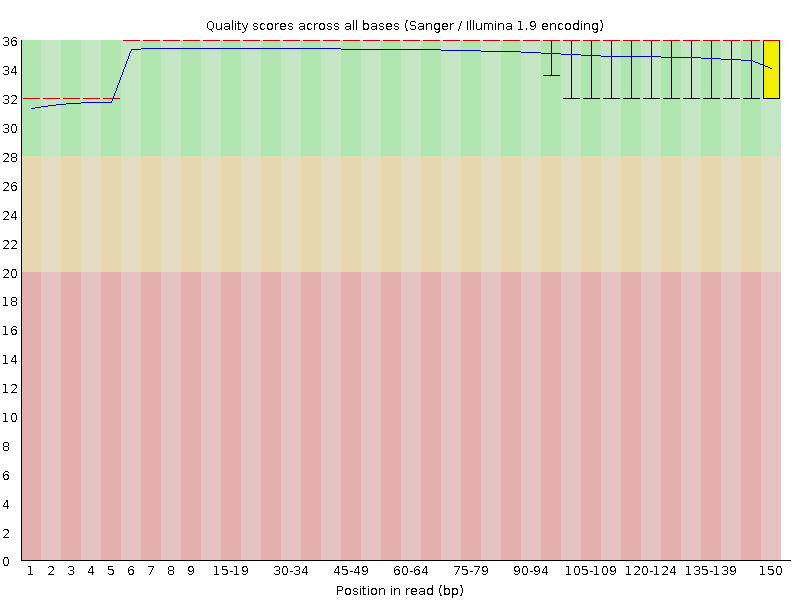

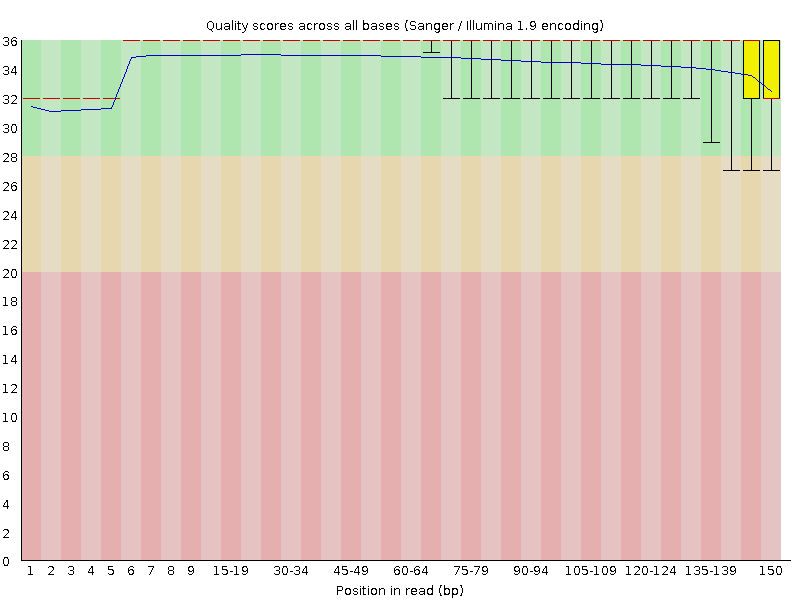


D. FastQC IS Reverse

C. FastQC IS Forward


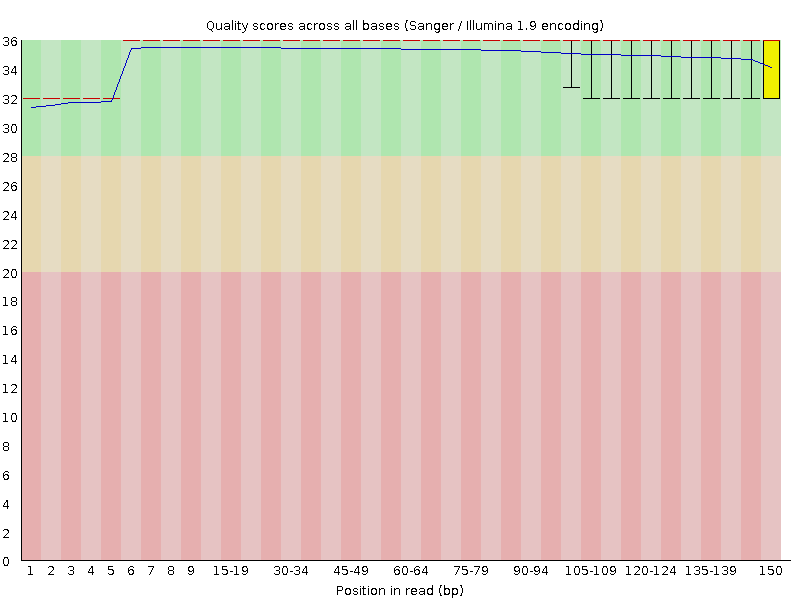

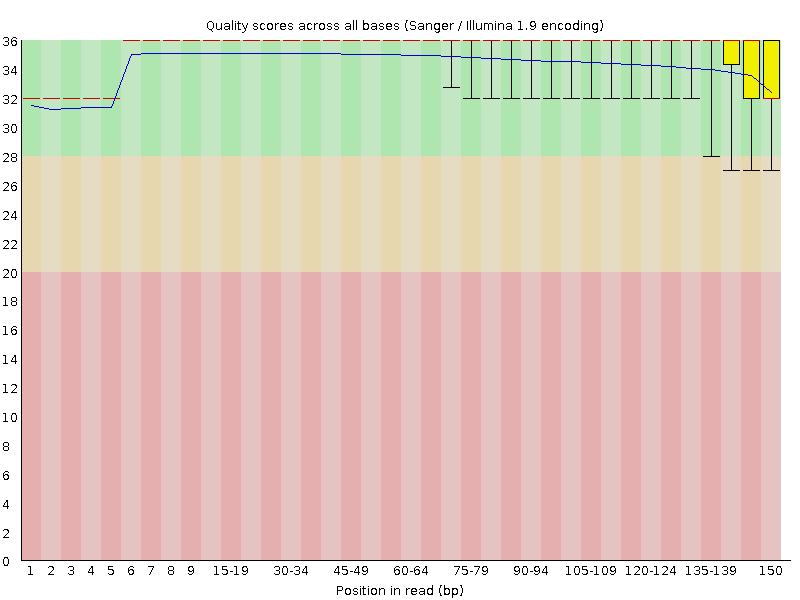


F. FastQC NIR Reverse

E. FastQC NIR Forward


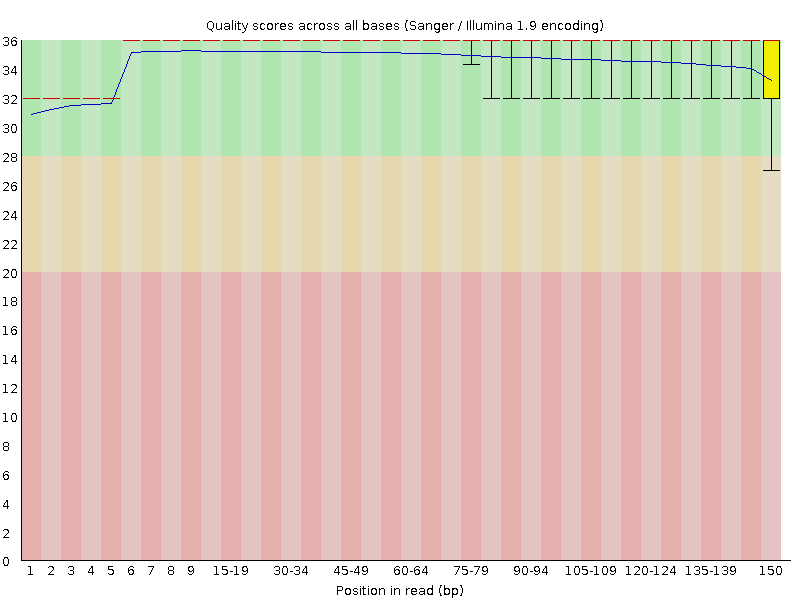

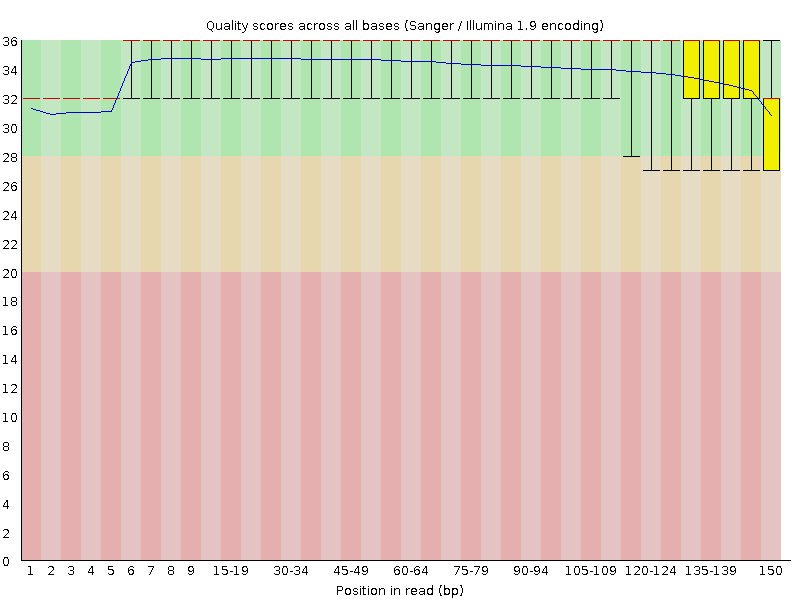


H. FastQC NIS Reverse

G. FastQC NIS Forward
